# Managing Orbital Floor Fractures With Three-Dimensional (3D)-Printed Surgical Guides: A Narrative Review

**DOI:** 10.7759/cureus.105823

**Published:** 2026-03-25

**Authors:** Vaibhav Anand, Mohit B Bagde, Sameer Gupta, Hitesh Patel, Niraj Kumar Yadav, G Harsha Vardhan Reddy, Omkar Eswara Babu Danda

**Affiliations:** 1 Department of Dentistry, Uttar Pradesh University of Medical Sciences, Saifai, IND; 2 Department of Head and Neck Surgery, Pandit Bhagwat Dayal Sharma Post Graduate Institute of Medical Sciences (PGIMS), Rohtak, IND; 3 Department of Oral and Maxillofacial Surgery, Inderprastha Dental College and Hospital, Ghaziabad, IND; 4 Department of Plastic and Reconstructive Surgery, Byramjee Jeejeebhoy Medical College, Ahmedabad, IND; 5 Department of Ophthalmology, Dr. Kailash Narayan Singh (KNS) Memorial Institute of Medical Sciences, Barabanki, IND; 6 Department of Hepato-Pancreato-Biliary Surgery (HPB) and Liver Transplantation, Institute of Liver and Biliary Sciences, New Delhi, IND; 7 Department of Conservative Dentistry and Endodontics, Dr. N. T. Rama Rao (NTR) University of Health Sciences, Vijayawada, IND

**Keywords:** orbital floor fracture, orbital reconstruction, patient-specific surgical guide, three-dimensional printing, virtual surgical planning

## Abstract

Orbital floor fractures are a common consequence of maxillofacial trauma and can result in significant functional and esthetic morbidity, including diplopia, enophthalmos, and impaired ocular motility if not accurately reconstructed. Conventional freehand orbital floor reconstruction relies heavily on intraoperative judgment and visual estimation, which may compromise precision, particularly in complex defects involving posterior orbital anatomy. The objective of this review is to evaluate the clinical effectiveness, accuracy, and feasibility of three-dimensional (3D)-printed patient-specific surgical guides in the management of orbital floor fractures. A comprehensive literature review was conducted using major electronic databases to identify prospective and comparative clinical studies assessing guide-assisted orbital reconstruction, with an emphasis on surgical accuracy, functional outcomes, complication rates, and workflow efficiency. The reviewed evidence demonstrates that 3D-printed surgical guides enable more precise implant positioning and improved restoration of orbital volume by translating virtual preoperative planning into reproducible intraoperative execution. Radiological assessments consistently show reduced discrepancies between reconstructed and contralateral orbits, while clinical outcomes indicate lower rates of postoperative diplopia, enophthalmos, and need for revision surgery compared with conventional techniques. 3D-printed surgical guides represent a promising adjunct in orbital floor fracture management, offering enhanced precision, predictability, and improved functional and esthetic outcomes, although further standardized and cost-effectiveness studies are warranted.

## Introduction and background

Orbital floor fractures are an established and clinically significant group of maxillofacial traumas, which are regularly linked to blunt facial trauma due to interpersonal violence, road traffic accidents, falls, and sporting activities [1]. Orbital floor disruption impairs orbital support and volume, resulting in functional impairments like diplopia, enophthalmos with restriction of extraocular movements, infraorbital nerve sensory disturbance, and observable facial asymmetry [2]. Poor reconstruction can lead to morbidity that persists and causes significant effects on visual ability and quality of life [3]. The surgical intervention aims to restore orbital anatomy and volume and to avoid long-term functional and aesthetic consequences [4]. Traditional orbital floor fracture treatment involves open reduction with internal reconstruction using manually shaped alloplastic implants via transconjunctival or subciliary incisions [5]. The placement of implants is greatly influenced by intraoperative experience and visual evaluation, without a clear reference to the preinjury anatomy of the orbit [6]. This subjective method adds to variations in the accuracy of surgery and postsurgical outcomes. Even the slightest mistakes in the implant placement or contouring can be manifested in clinically significant complications, especially delayed enophthalmos and chronic diplopia [7]. The complicated geometries of the orbit, including the lack of surgical exposure and the complex three-dimensional (3D) geometry of the orbit, make the reconstruction more complex than usual and highlight the shortcomings of the traditional freehand method [8].

Increased capacity of the preoperative evaluation of orbital defects was related to the advancements in maxillofacial imaging and computer-generated surgical planning [9]. Computed tomography (CT) is a high-resolution technique that allows proper delineation of fracture morphology, size of defects, and herniation of orbital contents [2]. These functions have been further expanded by virtual surgical planning to enable preoperative simulation of reconstruction onto contralateral mirrored anatomy [10]. Virtual surgical planning has been increasingly applied across maxillofacial reconstruction workflows, supporting clinically translatable digital planning for anatomically complex defects [11]. Digital planning has been expanded to 3D printing technology to promote the translation of virtual models to physical representations, including patient-specific surgical guides, providing a physical interface between preoperative planning and intraoperative implementation [11]. This imaging-to-physical translation is a critical step in maintaining reconstruction accuracy and workflow reliability across digital modeling and operative execution [12]. Surgical guides produced using 3D printing are designed to aid accurate positioning of the implant relative to predetermined anatomical landmarks based on patient-specific imaging data [5]. Guide-assisted methods can be used in orbital floor reconstruction to recover orbital volume and contour at increased accuracy by reproducing the anatomy of the normal orbit [12]. Efforts on reported clinical outcomes have not only covered prospective cohorts and comparative studies but also covered the following: better accuracy of implant positioning, better symmetry in postoperative imaging, less intraoperative alterations of implants, and reduced operative times [8]. Postoperative CT-based quantitative evaluations have shown improved concordance of intended vs. actual orbital volume restoration with guide-assisted reconstruction [13]. The use of functional outcomes (resolution of diplopia, decreased enophthalmos, and better ocular motility) has also been characterized with progressively growing frequency [14].

Although there is growing clinical interest, the incorporation of 3D-printed surgical guides into standard care for orbital trauma remains inconsistent. Variability in design approach, printing resources, fabrication accuracy, sterilization, and surgical workflow makes published studies heterogeneous. Accessibility is affected by economic factors, logistical limitations, and the need to use technical skills in virtual planning, especially in resource-scarce situations. There is a lack of uniformity in outcome reporting and minimal standardization of measures of accuracy, functional assessment instruments, and duration of follow-up. These factors make it difficult to compare studies directly, and they may limit the interpretation of clinical benefit across different centers. The increasing amount of future clinical evidence is favorable to the potential usefulness of 3D-printed surgical guides, but the synthesis toward clinical usefulness is skimpy. An organized analysis of the existing data is needed to clarify the indications, determine the credibility of the results, and establish useful factors applicable to maxillofacial surgeons and orbital experts. A focus on precision of reconstruction, restoration of function, complication rates, operating efficiency, and feasibility is consistent with current requirements of patient-centered surgical practice and the educational goals of clinically oriented medical academic journals. Practical limitations in digital workflows remain important determinants of clinical uptake, particularly the time required for segmentation and guide design, availability of in-house printing vs. outsourcing, sterilization requirements, and intraoperative fit verification. These workflow constraints may affect feasibility in emergency trauma settings and contribute to variation in reported outcomes between the centers.

The objective of this narrative review is to critically analyze prospective and comparative clinical evidence on computer-aided design (CAD)-assisted orbital floor fracture reconstruction using 3D-printed patient-specific surgical guides, compared with conventional freehand techniques. Emphasis is placed on radiologic accuracy, functional and esthetic outcomes, complication profiles, operative efficiency, and feasibility within routine clinical practice. Studies published between January 2015 and December 2025 were considered.

## Review

Methodology

Literature Search Strategy

This study was a narrative review synthesizing clinically relevant evidence on 3D-printed patient-specific surgical guides for orbital floor fracture reconstruction. A structured search of electronic databases, including PubMed, Scopus, Web of Science, and Google Scholar, was conducted to identify publications from January 2015 to December 2025.

Search terms combined controlled vocabulary and free-text keywords related to orbital floor fracture, blowout fracture, orbital reconstruction, 3D printing, patient-specific surgical guides, virtual surgical planning, and CAD/computer-aided manufacturing (CAM) technology. The PubMed search strategy used Boolean operators as follows: (“orbital floor fracture” OR “blowout fracture” OR “orbital reconstruction”) AND (“3D printing” OR “three-dimensional printing” OR “patient-specific” OR “surgical guide” OR “virtual surgical planning” OR “CAD/CAM”). Manual screening of reference lists from selected articles was also undertaken to identify additional relevant publications. Only English-language studies involving human subjects were included to ensure clinical applicability.

In addition to primary clinical studies, systematic reviews and comparative trials evaluating virtual planning workflows vs. conventional reconstruction were actively sought to contextualize workflow validation and clinical translation.

Although this review does not claim systematic review methodology, the search strategy was designed to capture representative contemporary comparative evidence to minimize the omission of relevant virtual planning studies.

Selection Approach

As this review follows a narrative design, study selection aimed to identify clinically informative and methodologically relevant evidence rather than to generate a statistically pooled dataset. Titles and abstracts were screened for relevance to guide-assisted orbital floor reconstruction, and full texts were reviewed when studies reported radiologic, functional, esthetic, workflow, or feasibility outcomes related to virtual planning or 3D-printed surgical guides.

Eligible publications included prospective studies, retrospective comparative studies, cohort studies, clinical case series, and relevant systematic reviews examining digital planning workflows or patient-specific implant guidance in orbital reconstruction. Cadaveric investigations, purely technical engineering reports without clinical outcomes, single case reports, conference abstracts, and studies addressing nontraumatic orbital reconstruction were excluded.

Data Considerations and Evidence Contextualization

Given the narrative nature of this review, formal quantitative data-extraction templates were not used. Instead, methodological characteristics, workflow descriptions, and outcome domains were examined to allow thematic comparison across studies. Particular emphasis was placed on radiologic accuracy, assessed through CT-based volumetric analysis and implant position concordance; functional recovery, including diplopia resolution and ocular motility; esthetic restoration, including globe symmetry and enophthalmos correction; complication rates; revision surgery; and operative efficiency.

Digital Imaging and Workflow Context

Most included studies utilized CT datasets for defect assessment and contralateral mirroring in virtual surgical planning. Preoperative data acquisition and digital model accuracy are critical determinants of guide reliability, particularly when integrating CT-derived segmentation with adjunct surface-scanning workflows, such as intraoral or extraoral scanning [12]. Guide design generally relied on patient-specific segmentation and landmark-based positioning principles to reproduce preinjury orbital contour and volume. Postoperative evaluation was primarily performed using CT-based volumetric comparison between planned and achieved reconstructions.

The clinical translation of virtual surgical planning workflows has been evaluated in broader maxillofacial reconstruction literature, demonstrating improved reproducibility, planning precision, and operative predictability when digital modeling is integrated into surgical execution [11]. The inclusion of this evidence supports the link between workflow validation and clinical applicability in orbital reconstruction.

Narrative Synthesis Framework

This review adopts a qualitative and thematic synthesis approach rather than a systematic meta-analytic methodology. Meta-analysis was not performed due to substantial heterogeneity in study designs, outcome measures, guide fabrication protocols, and follow-up durations, which precluded meaningful quantitative pooling. Formal Preferred Reporting Items for Systematic reviews and Meta-Analyses flow diagrams, protocol registration, and structured risk-of-bias scoring were not undertaken. The objective was to critically interpret contemporary clinical and comparative evidence to inform surgical practice.

Epidemiology and clinical significance of orbital floor fractures

Fractures of the orbital floor constitute a significant proportion of maxillofacial trauma presenting to emergency and trauma services [15]. According to epidemiological data, young adult men are more frequently affected, reflecting increased exposure to high-energy mechanisms including interpersonal violence, road traffic accidents, occupational hazards, and contact sports [6]. Falls are particularly prevalent in pediatric and elderly populations, where age-related anatomical and physiological factors influence fracture pattern and clinical presentation [16]. The orbital floor is structurally thin and vulnerable to fracture, especially with sudden increases in intraorbital pressure or when force is transmitted directly through the orbital rim [17]. Orbital floor fractures have clinical implications beyond skeletal disruption and may result in functional, neurological, and esthetic consequences. Displacement of orbital contents into the maxillary sinus can lead to diplopia, limitation of extraocular muscle movement, and displacement of the globe position [9]. Involvement of the infraorbital nerve commonly causes sensory disturbances in the cheek, upper lip, and maxillary dentition, contributing to patient discomfort and functional impairment [18]. Delayed enophthalmos and hypoglobus remain among the most concerning sequelae and may develop weeks after injury due to a progressive increase in orbital volume [10].

**Figure 1:**
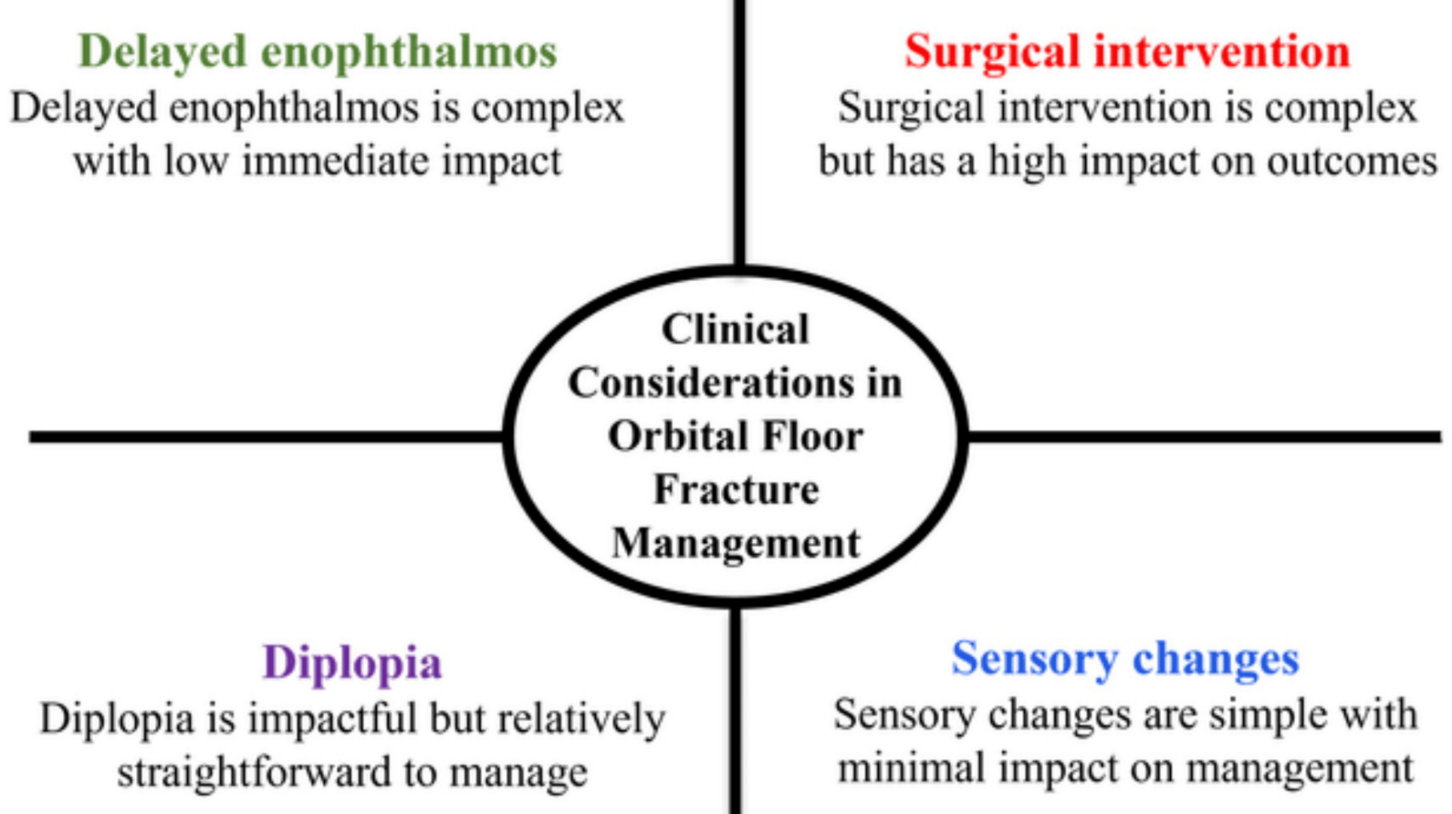
Determinants affecting management decisions in orbital floor fractures Image credit: This is an original image created by the author Vaibhav Anand

Timely and accurate diagnosis is critical for optimizing outcomes. CT is the preferred imaging modality for accurately determining fracture extent, defect size, soft-tissue entrapment, and orbital volume change [19]. Factors influencing clinical decision-making regarding surgical intervention include symptom severity, radiologic evidence, and the likelihood of late deformity [7]. Poor or delayed reconstruction may be associated with persistent diplopia, facial asymmetry, and reduced quality of life, highlighting the importance of appropriate management strategies [20]. From a broader healthcare perspective, orbital floor fractures also impose economic and psychosocial burdens. Occupational performance and social interaction may be negatively affected by prolonged visual impairment, esthetic deformity, and sensory loss [13]. Identification of epidemiological patterns and clinical implications provides a foundation for refining surgical interventions to improve precision, predictability, and long-term patient outcomes [8]. Figure 1 illustrates the key clinical factors influencing decision-making in the management of orbital floor fractures.

Anatomy of the orbital floor and biomechanics of fracture

The orbital floor forms the inferior boundary of the orbit and separates the orbital contents from the maxillary sinus [5]. The bony floor is composed predominantly of the maxilla, with contributions from the zygomatic and palatine bones [18]. Its relative thinness, particularly in the posterior and medial regions, increases susceptibility to fracture under traumatic stress [20]. The concave configuration and posterior slope influence force dissipation and contribute to the characteristic distribution of fractures following blunt impact [21]. Several vital neurovascular and muscular structures lie in close proximity to the orbital floor. The infraorbital nerve courses along the infraorbital groove and canal and supplies sensation to the midface [22]. The inferior rectus and inferior oblique muscles lie directly above the floor and are supported by orbital fat and periosteum [17]. Disruption or displacement of these structures following fracture contributes to ocular motility restriction, diplopia, and alteration in globe position. Anatomical variation in sinus pneumatization, bony thickness, and orbital volume may further influence individual susceptibility to injury [11].

Two complementary biomechanical mechanisms are commonly used to explain orbital floor fractures [5]. The hydraulic mechanism involves a rapid increase in intraorbital pressure following blunt impact to the globe, with transmission of force to the thin orbital floor resulting in fracture [23]. The buckling mechanism describes the transmission of forces from the orbital rim to the floor following direct facial impact, leading to structural failure without direct compression of the globe [24]. These mechanisms frequently coexist, producing complex fracture patterns with variable defect size and herniation of orbital soft tissues. Fracture morphology is influenced by impact direction, energy magnitude, and anatomical constraints [12].

Posteromedial defects are associated with a higher likelihood of muscle entrapment and orbital volume expansion, whereas anterior defects may demonstrate minimal displacement [16]. Accurate understanding of orbital anatomy and fracture biomechanics is essential for reconstructive planning, as restoration of orbital volume and contour depends on precise evaluation of defect geometry and adjacent structures [24]. The limitations of subjective intraoperative estimation highlight the value of modern imaging and 3D planning in contemporary orbital reconstruction [10]. Key anatomical elements and biomechanical factors influencing fracture patterns and clinical presentation are summarized in Table 1.

**Table 1:** Orbital floor anatomy, biomechanics, and clinical implications Source: Compiled by the authors based on information synthesized from [9,14,16,21,22]. The table is not directly reproduced from any single external source; therefore, permission from the original publishers was not required

Anatomical component	Location	Structural characteristics	Clinical relevance	Injury implications	Reference
Orbital floor	Inferior orbit	Thin maxillary bone, concave	Globe support	High fracture susceptibility	[14]
Infraorbital nerve	Infraorbital canal	Sensory neural pathway	Midfacial sensation	Paresthesia, hypoesthesia	[22]
Inferior rectus muscle	Superior to the floor	Extraocular muscle	Ocular motility	Diplopia, entrapment	[16]
Maxillary sinus	Inferior to the floor	Pneumatized cavity	Pressure modulation	Herniation of contents	[9]
Orbital fat	Throughout orbit	Adipose cushioning tissue	Volume maintenance	Globe displacement	[21]

Conventional surgical approaches to orbital floor reconstruction

The surgical management of orbital floor fractures traditionally aims to restore orbital anatomy, reestablish orbital volume, and prevent functional and aesthetic complications [20]. Surgical intervention is generally indicated in cases of persistent diplopia, significant enophthalmos, muscle entrapment, or large bony defects identified on imaging [14]. Conventional techniques rely on direct exposure of the orbital floor followed by placement of an alloplastic implant to support herniated orbital contents. Access is most commonly achieved through transconjunctival or subciliary incisions [24]. The transconjunctival approach offers favorable cosmetic outcomes with minimal visible scarring and adequate exposure of the inferior orbit, making it suitable for most clinical cases [13]. Subciliary and subtarsal approaches may provide wider surgical access but are associated with a higher risk of lower eyelid malposition and cutaneous scarring [7]. The choice of approach depends on fracture characteristics, associated facial injuries, and surgeon preference.

Reconstruction involves placement of either preshaped or manually contoured implants composed of titanium mesh, porous polyethylene, or resorbable polymers [3]. Implant positioning is performed intraoperatively under visual and tactile guidance, with contouring based on perceived restoration of orbital floor continuity [16]. This reliance on intraoperative estimation introduces variability in implant positioning, contour accuracy, and defect coverage, particularly in posterior or complex fractures [24]. Traditional intraoperative assessment of reconstruction adequacy is limited to forced duction testing, visual inspection, and estimation of globe position. Minor discrepancies in orbital volume restoration may remain undetected until postoperative imaging or delayed clinical presentation [25]. Reported complications associated with conventional methods include residual diplopia, enophthalmos, implant malposition, infraorbital nerve dysfunction, and the need for secondary revision surgery [14].

Although conventional orbital floor reconstruction techniques are well-established and widely practiced, inherent limitations in reproducibility and precision remain. Dependence on subjective intraoperative judgment and restricted visualization of posterior orbital anatomy contribute to variability in clinical outcomes. Recognition of these limitations has prompted growing interest in adjunctive technologies to improve anatomical accuracy, reduce intraoperative uncertainty, and enhance predictability in orbital trauma reconstruction.

Principles of 3D printing in maxillofacial surgery

Additive manufacturing, also known as 3D printing, has emerged as an important technology in maxillofacial surgery due to its ability to translate digital imaging data into patient-specific physical models [26]. High-resolution CT or cone-beam CT datasets are processed using specialized software to generate 3D craniofacial virtual models that closely reproduce patient anatomy [21]. These digital reconstructions allow detailed visualization of complex anatomical relationships and pathological deformities, thereby improving diagnostic precision and preoperative planning [27]. 3D printing is used to fabricate anatomical models, surgical guides, splints, and customized implants in maxillofacial trauma and reconstruction [19]. Physical models provide haptic and spatial representation of fracture patterns and defect morphology, facilitating preoperative simulation and enhancing surgical preparedness [23]. Surgical guides enable translation of virtual planning into intraoperative reference tools that support controlled osteotomies, accurate implant placement, and predictable restoration of anatomical contours [28]. Customization to individual anatomy reduces reliance on intraoperative estimation and enhances procedural precision.

The printing process involves layer-by-layer material deposition using technologies such as stereolithography, selective laser sintering, fused deposition modeling, and digital light processing [25]. The selection of printing technique and material depends on the intended clinical application, required dimensional accuracy, mechanical strength, and compatibility with sterilization protocols. Photopolymer resins, thermoplastics, and biocompatible polymers are commonly used materials [13]. Integration of 3D printing into maxillofacial workflows has been associated with reduced operative time, improved reconstruction accuracy, and enhanced procedural consistency [4]. Guide-assisted implant placement and preshaped implant designs minimize intraoperative manipulation and improve conformity with preoperative planning [18]. Quantitative studies have demonstrated closer correspondence between planned and achieved anatomical reconstruction when additive manufacturing techniques are incorporated into surgical procedures [26].

Despite demonstrated benefits, implementation remains influenced by cost, technical expertise, production time, and institutional infrastructure. Workflow standardization and validation of accuracy metrics continue to evolve [11]. Ongoing advancements in software automation, in-house printing capabilities, and the development of biocompatible materials are expected to enhance accessibility and clinical integration of 3D printing in maxillofacial surgery. Table 2 summarizes key aspects of 3D printing processes and their clinical applications.

**Table 2:** Three-dimensional printing in maxillofacial surgery Source: Compiled by the authors based on information synthesized from [12,19,21,23,27]. The table is not directly reproduced from any single external source; therefore, permission from original publishers was not required CT: computed tomography, CBCT: cone-beam computed tomography, SLA: stereolithography, SLS: selective laser sintering, FDM: fused deposition modeling, DLP: digital light processing

Aspect	Description	Common techniques	Clinical application	Reference
Imaging data	Digital radiological input	CT, CBCT	Anatomical reconstruction	[27]
Printing technology	Layer-by-layer fabrication	SLA, SLS, FDM, DLP	Models, guides, implants	[23]
Materials	Printable substrates	Resins, polymers	Surgical accuracy	[12]
Surgical guides	Patient-specific tools	Virtual planning-based	Implant positioning	[21]
Workflow integration	Preoperative to intraoperative	Digital-to-physical transfer	Outcome predictability	[19]

Design and fabrication of 3D-printed surgical guides

The design and fabrication of 3D-printed surgical guides are based on integration of high-resolution imaging, virtual surgical planning, and additive manufacturing technologies [29]. Anatomical reconstruction is typically performed using CT or cone-beam CT datasets, which allow delineation of fracture margins, defect geometry, and structures surrounding the orbital volume [30]. Digital imaging data are imported into specialized planning software for segmentation and surface modeling, generating a 3D representation of patient-specific anatomy [5]. Virtual surgical planning enables simulation of fracture reduction and implant positioning with the goal of restoring native orbital contours [19]. In orbital floor reconstruction, contralateral mirroring of the intact orbit is commonly used to estimate optimal implant curvature and positioning [25]. Guide design incorporates predefined contact sites on stable bony landmarks to facilitate reproducible intraoperative seating and stability. Consideration of guide thickness, edge configuration, and soft tissue clearance improves surgical usability and reduces interference with implant placement [29].

Guide fabrication is typically performed using additive manufacturing methods such as stereolithography, selective laser sintering, and fused deposition modeling [30]. The selection of printing technology depends on the dimensional accuracy required, mechanical strength, and compatibility with sterilization protocols [31]. Photopolymer resins and medical-grade thermoplastics are widely used due to their favorable resolution and intraoperative handling properties [7]. Postprocessing steps include removal of support structures, surface finishing, and dimensional verification to maintain fidelity to the virtual design [18]. Low-temperature sterilization methods, including hydrogen peroxide plasma or ethylene oxide, are commonly required for clinical application [26]. Quality control measures focus on dimensional accuracy, surface smoothness, and structural stability under operative conditions. Errors in imaging segmentation, surface modeling, or fabrication may translate into inaccurate guide fit and reduced reliability of implant positioning, thereby undermining the intended advantages of guided reconstruction [19]. Workflow standardization and validation of accuracy metrics continue to evolve and remain important for broader clinical adoption of patient-specific surgical guides in orbital trauma care [30].

Surgical workflow using 3D-printed guides for orbital floor repair

The surgical workflow incorporating 3D-printed guides for orbital floor reconstruction begins with comprehensive preoperative imaging and virtual surgical planning [27]. The extent of fracture, defect geometry, and orbital volume mismatch are defined using high-resolution CT datasets and compared with the contralateral orbit [32]. Virtual planning enables preoperative simulation of implant position and contour modification, generating a predetermined reconstructive plan that guides intraoperative execution [17]. Following virtual planning, patient-specific surgical guides are designed to align with stable bony landmarks of the orbit and infraorbital region [26]. These guides provide reproducible seating and orientation during surgery, thereby translating preoperative planning into intraoperative reference points [33]. Preoperative verification of guide fit using anatomical models or digital simulation is commonly performed to enhance reliability and reduce unexpected intraoperative modification [20].

**Figure 2:**
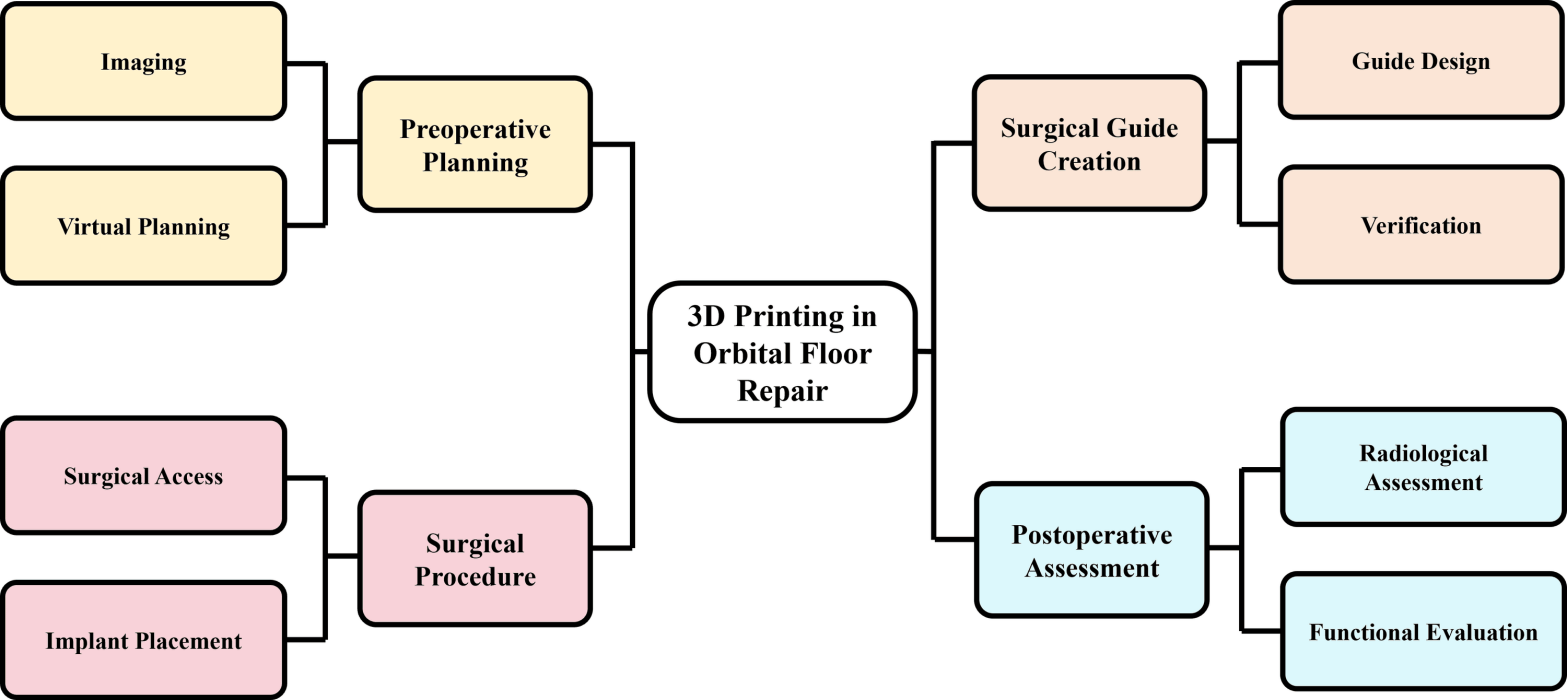
Workflow of 3D printing-assisted orbital floor repair 3D: three-dimensional Image credit: This is an original image created by the author Vaibhav Anand

Surgical access to the orbital floor is typically achieved through established approaches such as transconjunctival or subtarsal incisions. After exposure and reduction of herniated orbital contents, the guide is positioned on predetermined anatomical surfaces [18]. Accurate guide seating facilitates controlled placement of the orbital implant, supporting compliance with the planned reconstruction parameters [34]. Guide-assisted positioning reduces reliance on visual estimation and tactile judgment, particularly in posterior or medial defects where intraoperative visualization is limited [21]. After implant placement, the guide is removed, and implant stability is assessed. Intraoperative forced duction testing and visual examination are performed to confirm restoration of ocular mobility and appropriate globe position [29]. Reduced need for implant contouring or repositioning may improve procedural efficiency and minimize soft tissue manipulation. Postoperative radiologic assessment is performed to evaluate concordance between planned and achieved reconstruction [11]. Comparative studies have reported improved contour matching and orbital volume restoration with guided workflows compared with conventional freehand techniques [19]. Overall, integration of 3D-printed guides into orbital floor repair is intended to enhance accuracy, reproducibility, and predictability, with potential improvement in functional and aesthetic outcomes in appropriately selected cases [8]. Figure 2 illustrates the clinical workflow for incorporating 3D printing into orbital floor reconstruction.

Clinical outcomes and accuracy of reconstruction

Guide-assisted orbital floor reconstruction has been increasingly evaluated with respect to objective measures of anatomical accuracy and postoperative functional restoration [34]. 3D-printed patient-specific surgical guides facilitate translation of preoperative virtual planning into intraoperative execution aimed at restoring orbital contour and volume [27]. Quantitative radiologic assessments using postoperative CT have demonstrated greater concordance between planned and achieved implant positioning with guided techniques, particularly in posterior and medial defects where visualization is limited [14]. Orbital volume restoration is a critical determinant of long-term functional and esthetic outcomes [30]. Volumetric analyses indicate reduced discrepancies between reconstructed and contralateral orbits in guide-assisted reconstruction, reflecting improved anatomical symmetry [35]. Accurate orbital volume restoration is associated with a reduced incidence of delayed enophthalmos and hypoglobus, complications frequently attributed to underestimating volume in freehand reconstruction [28]. Improved spatial control of implant positioning reduces the likelihood of overcorrection or undercorrection and enhances reconstructive predictability.

Enhanced positioning accuracy is also reflected in reduced need for intraoperative implant adjustment and lower revision rates. Guide-assisted procedures enable the seating of implants along predefined anatomical planes, thereby decreasing rotational and translational errors [36]. Postoperative imaging commonly confirms improved adaptation of implants to defect margins with minimal gaps or malposition. These advantages are particularly relevant in complex fractures with extensive defects or irregular geometries [30]. Functional outcomes following guided reconstruction show favorable trends. Reported rates of postoperative diplopia and extraocular movement restriction are reduced, likely due to improved stabilization of orbital contents and minimized soft tissue impingement [16]. Sensory recovery following infraorbital nerve involvement has been reported to be comparable or improved, potentially related to reduced manipulation and more controlled implant placement [36]. Patient-reported satisfaction regarding esthetic symmetry and ocular comfort has also been shown to improve with guide-assisted techniques [31].

Comparative clinical studies have generally demonstrated equal or superior radiologic accuracy and functional recovery with guide-assisted reconstruction compared with conventional freehand techniques, while maintaining comparable complication profiles. Recent systematic reviews evaluating virtual planning workflows have similarly reported improved implant positioning precision and orbital volume restoration when digital planning and 3D-printed guides are integrated into reconstruction, although heterogeneity in study design, outcome metrics, and follow-up duration limits direct quantitative comparison.

Despite these favorable findings, variability persists across studies due to differences in assessment protocols, follow-up duration, and guide design methodology [33]. Standardization of volumetric accuracy measures and functional outcome assessment tools remains necessary to enable meaningful interstudy comparison [26]. The current body of evidence supports the role of 3D-printed surgical guides in enhancing the precision and reproducibility of orbital floor reconstruction as an adjunct to conventional techniques [19]. Implementation feasibility, however, is influenced by access to specialized planning software, trained personnel, and additive manufacturing infrastructure, which may limit adoption in resource-constrained environments and urgent trauma settings. Table 3 summarizes the comparative clinical and radiologic outcomes of guide-assisted and conventional orbital floor reconstruction.

**Table 3:** Clinical outcomes and reconstruction accuracy Source: Compiled by the authors based on information synthesized from [21,28,30,31,34]. The table is not directly reproduced from any single external source; therefore, permission from original publishers was not required CT: computed tomography

Outcome parameter	Assessment method	Guide-assisted reconstruction	Conventional reconstruction	Reference
Orbital volume restoration	CT-based volumetry	High accuracy, minimal discrepancy	Variable accuracy	[30]
Implant positioning	Postoperative CT	Precise alignment with planning	Subjective alignment	[34]
Diplopia resolution	Clinical examination	Reduced incidence	Higher persistence	[28]
Enophthalmos rate	Clinical measurement	Lower occurrence	Increased risk	[21]
Revision surgery	Follow-up analysis	Reduced need	Higher frequency	[31]

Functional and esthetic outcomes

Functional and esthetic outcomes represent critical endpoints in orbital floor fracture management because they reflect both anatomical restoration and patient recovery [30]. Proper reconstruction of the orbital floor is essential for preservation of globe position, extraocular muscle function, and facial symmetry. Surgical guides created using 3D printing have been reported to enhance implant positioning accuracy and improve control of orbital volume restoration, thereby increasing predictability of these outcomes [37]. Functional recovery is typically assessed through resolution of diplopia, restoration of ocular motility, and improvement of infraorbital sensory symptoms. Guide-assisted reconstruction supports repositioning of herniated orbital contents and may reduce the risk of muscle impingement or malposition [38]. Clinically, guided techniques have been associated with lower persistence of postoperative diplopia and improved extraocular movement, particularly in fractures involving posterior or medial defects [34]. Infraorbital nerve recovery has also shown favorable trends, and reduced long-term hypoesthesia has been attributed to decreased intraoperative manipulation [27].

Esthetic outcomes are closely linked to the restoration of orbital contour and globe projection. Inadequate orbital volume restoration may lead to delayed enophthalmos, hypoglobus, and facial asymmetry, often becoming apparent weeks after injury [38]. Guide-assisted workflows improve volumetric consistency and may enhance globe projection symmetry compared with the contralateral orbit [18]. Postoperative imaging studies have shown closer symmetry indices and reduced volumetric discrepancy when patient-specific guides are used [7]. The contribution of guided reconstruction to patient-perceived facial appearance and ocular comfort is increasingly recognized, particularly as patient-reported outcomes are reported more frequently. Accurate anatomical restoration has been associated with improved confidence in facial symmetry, reduced visual strain, and improved quality of life [11]. A reduced need for secondary corrective procedures may further support the esthetic reliability of guided reconstruction.

Despite these favorable trends, variability in the assessment of functional and aesthetic outcomes remains common across studies. Differences in follow-up duration, evaluation methods, and reporting standards limit direct comparison [21]. Further prospective evidence using standardized functional and esthetic outcome measures is required to clarify long-term benefit and improve comparability across clinical settings.

Complications, limitations, and cost considerations

Orbital floor reconstruction using 3D-printed surgical guides has demonstrated favorable safety profiles, with complication rates reported to be comparable to those of conventional methods [39]. Common postoperative complications include diplopia, implant malposition, infection, and infraorbital nerve sensory disturbance, although these outcomes are generally uncommon and are often more closely related to fracture severity than to guide usage itself [34]. Proper guide seating and adherence to planned reconstruction parameters may reduce the likelihood of implant displacement and soft tissue impingement [27].

Despite reported clinical benefits, several limitations influence broader adoption. Advanced imaging, segmentation, and virtual modeling are required during preoperative planning, which increases preparation time before surgery [37]. Time constraints and fabrication delays may restrict feasibility in acute trauma settings where urgent intervention is required. In addition, guide fit may be compromised by anatomical distortion caused by edema, comminution, or extensive soft-tissue injury, potentially reducing intraoperative accuracy in selected cases [39].

Technical expertise and infrastructure requirements represent additional barriers. Access to specialized software, trained personnel, and additive manufacturing facilities is uneven across institutions [40]. Variability in guide design protocols, printing resolution, and material properties contributes to heterogeneity in reported outcomes. The absence of universal standards for validating guide design and measuring accuracy further complicates comparison across clinical settings [18]. Economic factors also influence clinical decision-making. Costs related to imaging, virtual planning, software licensing, printing materials, and sterilization may increase overall treatment expenditure [36]. This limitation is particularly relevant in resource-constrained environments where conventional reconstruction remains more accessible [19]. Cost-effectiveness studies suggest potential indirect savings due to reduced operative time, fewer revisions, and improved long-term outcomes; however, robust economic evidence remains limited [24].

Material-related limitations should also be considered. Some printable polymers are sensitive to high-temperature sterilization and require low-temperature sterilization methods, which may not be universally available [21]. Guides must be assessed for dimensional stability and structural integrity to ensure intraoperative reliability. Recognition of these complications and limitations supports appropriate case selection and optimization of workflow integration [38]. Continued developments in automation, material science, and in-house printing capacity may reduce existing barriers and improve cost-effectiveness, thereby supporting wider clinical implementation of guided orbital floor reconstruction [31].

Synthesis of current evidence

Accumulating clinical evidence indicates that 3D-printed surgical guides represent a valuable adjunct in orbital floor fracture reconstruction [40]. Prospective and comparative clinical studies have demonstrated greater consistency in implant positioning and improved accuracy in restoration of preinjury orbital anatomy when guided techniques are used [36]. Radiologic evaluations consistently report improved concordance between preoperative planning and postoperative reconstruction, particularly in complex fractures involving posterior or medial orbital regions [27]. Orbital volume restoration remains a principal determinant of functional and esthetic success [17]. Guide-assisted reconstruction has been associated with fewer cases of delayed enophthalmos and globe malposition compared with conventional freehand techniques, likely due to reduced volumetric discrepancy between affected and contralateral orbits [33]. Enhanced control of implant contour and seating contributes to reduced intraoperative variability and improved anatomical predictability [26].

Clinical outcome measures further support these findings. Guided reconstruction has been associated with improved ocular motility, reduced persistence of postoperative diplopia, and favorable sensory recovery, potentially reflecting improved support of orbital contents and reduced soft tissue impingement [39]. Patient-reported measures indicate improved satisfaction with facial symmetry and visual comfort, and greater anatomical accuracy appears to correlate with quality-of-life improvements [31].

Despite these favorable trends, heterogeneity across published studies remains substantial. Variations in study design, sample size, fabrication protocols, and outcome assessment methods limit direct comparability [25]. Follow-up duration differs considerably among studies, influencing the evaluation of delayed complications such as progressive enophthalmos or implant-related morbidity [22]. The absence of standardized accuracy metrics and uniform functional assessment tools continues to constrain high-level evidence synthesis.

The current literature supports the role of guide-assisted workflows in improving surgical precision and reproducibility by integrating patient-specific planning with intraoperative guidance [38]. However, further methodological refinement, standardized reporting frameworks, and consistent outcome measures are required to consolidate the evidence base and clarify the long-term clinical value of 3D-printed surgical guides in orbital floor reconstruction.

Limitations and future recommendations

A few limitations influence the interpretation of the current evidence on guide-assisted orbital floor reconstruction. Diversity in study design, sample size, and duration of follow-up restricts direct comparison across clinical series. Heterogeneity in guide design protocols, printing technologies, material properties, and accuracy assessment methods further contributes to variability in reported outcomes. Limited use of standardized volumetric and functional assessment tools complicates the synthesis of findings. Variability in reporting radiologic endpoints, definitions of successful orbital volume restoration, and timing of postoperative imaging also limits cross-study comparability. Potential selection bias toward complex fractures or elective cases may affect generalizability to broader trauma populations. Economic data remain limited, and formal cost-effectiveness analyses across different healthcare systems are sparse.

Future research should prioritize well-designed prospective and comparative trials with standardized imaging protocols, guide fabrication workflows, and outcome assessment criteria. Broader adoption of validated volumetric and functional evaluation tools would improve comparability and clinical interpretability. Uniform definitions of accuracy metrics, complication reporting, and follow-up intervals would facilitate higher level evidence synthesis. Multicenter collaboration may enhance sample diversity and external validity. Integration of economic evaluation alongside clinical outcomes is necessary to clarify cost-benefit profiles. Development of structured reporting frameworks specific to guide-assisted orbital reconstruction may further strengthen methodological consistency and support informed clinical implementation.

## Conclusions

Surgical guides that are 3D-printed have become a helpful addition to the treatment of orbital floor fractures and have shown greater precision, repeatability, and predictability in reconstruction. Patient-specific imaging, virtual planning, and guide-assisted implant placement can be integrated to provide accurate restoration of orbital anatomy and volume, thereby overcoming the major weaknesses of traditional freehand methods. Clinical evidence shows greater accuracy in implant positioning, smaller orbital volume mismatch, and positive functional and esthetic outcomes, such as less diplopia, enophthalmos, and revision surgery. Guide-controlled workflows help achieve efficiency in the process, as intraoperative changes to implants and the use of subjective evaluation are minimized. Although there is an inconsistency in design protocols, the fabrication processes, and outcome reporting, there are consistent tendencies toward enhanced anatomical quality and clinical reliability with guided reconstruction. The accessibility and adoption depend on factors such as cost, technical expertise, and infrastructure, especially in the acute trauma setting. Further development of planning processes, standardization and systematization of evaluation metrics, and inclusion in regular surgical practice can further increase clinical utility. Altogether, 3-D printing of surgical manuals is a clinically pertinent idea of dealing with orbital floor fractures as a way of reconstructing the orbital floor precisely and ensuring better patient outcomes and higher standards in surgery.
